# 3-(5-Oxo-3-phenyl-4,5-dihydro-1*H*-pyrazol-1-yl)benzonitrile

**DOI:** 10.1107/S1600536812027444

**Published:** 2012-06-27

**Authors:** Ling Li, Rong-sheng Tong, Jin-qi Li, Jian-you Shi

**Affiliations:** a Bioengineering College, Xihua University, Sichuan Provincial, People’s Hospital, Chengdu 610039, People’s Republic of China; b Department of Pharmacy, Sichuan Academy of Medical Science and Sichuan Provincial, People’s Hospital, Chengdu 610072, People’s Republic of China

## Abstract

In the title compound, C_16_H_11_N_3_O, the dihedral angles between the 3-cyano­benzene and benzene planes and the 1*H*-pyrazol-5(4*H*)-one plane are 4.97 (9) and 9.91 (9)°, respectively.

## Related literature

For a similar structure, see: Paulis *et al.* (2006 ▶).
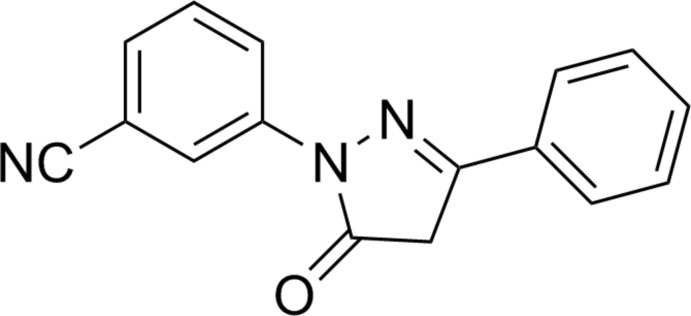


## Experimental

### 

#### Crystal data

C_16_H_11_N_3_O
*M* *_r_* = 261.28
Monoclinic, 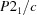

*a* = 7.6683 (3) Å
*b* = 17.8013 (7) Å
*c* = 9.7574 (4) Å
β = 106.506 (4)°
*V* = 1277.05 (9) Å^3^
*Z* = 4
Mo *K*α radiation
μ = 0.09 mm^−1^
*T* = 293 K
0.34 × 0.30 × 0.28 mm

#### Data collection

Agilent Xcalibur diffractometer with an Eos CCD detector
Absorption correction: multi-scan (*CrysAlis PRO*; Agilent, 2011 ▶) *T*_min_ = 0.967, *T*_max_ = 1.000
5145 measured reflections
2608 independent reflections
1686 reflections with *I* > 2σ(*I*)
*R*_int_ = 0.023

#### Refinement

*R*[*F* ^2^ > 2σ(*F* ^2^)] = 0.048
*wR*(*F* ^2^) = 0.119
*S* = 1.02
2608 reflections
182 parameters
H-atom parameters constrained
Δρ_max_ = 0.14 e Å^−3^
Δρ_min_ = −0.13 e Å^−3^

### 

Data collection: *CrysAlis PRO* (Agilent, 2011 ▶); cell refinement: *CrysAlis PRO*; data reduction: *CrysAlis PRO*; program(s) used to solve structure: *OLEX2.solve* (Bourhis *et al.*, 2012 ▶); program(s) used to refine structure: *SHELXL97* (Sheldrick, 2008 ▶); molecular graphics: *OLEX2* (Dolomanov *et al.*, 2009 ▶); software used to prepare material for publication: *OLEX2*.

## Supplementary Material

Crystal structure: contains datablock(s) I, global. DOI: 10.1107/S1600536812027444/zs2212sup1.cif

Structure factors: contains datablock(s) I. DOI: 10.1107/S1600536812027444/zs2212Isup2.hkl

Supplementary material file. DOI: 10.1107/S1600536812027444/zs2212Isup3.cml

Additional supplementary materials:  crystallographic information; 3D view; checkCIF report

## Figures and Tables

**Fig. 1.**
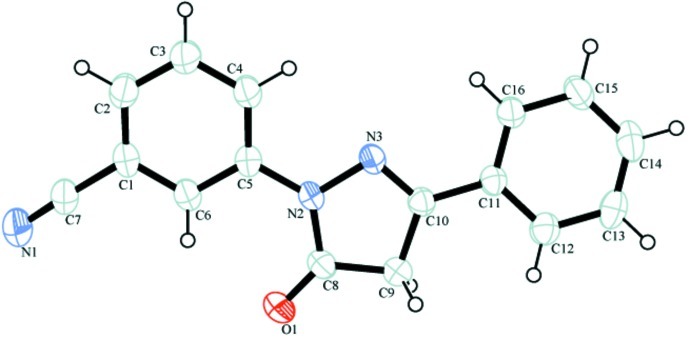
The molecular structure of title compound showing atom numbering, with displacement ellipsoids drawn at the 40% probability level.

## supplementary crystallographic information

### Comment

In our research, 1,3-Diphenyl-1*H*-pyrazol-5(4*H*)-one is a member of a series of compounds which are being investigated for their potential as anticancer agents. In the analogous title compound, C_16_H_11_N_3_O, (Fig. 1), the dihedral angles between the 3-cyanobenzene and benzene planes and the 1*H*-pyrazol-5(4)-one plane are 4.97 (9)° and 9.91 (9)°, respectively. Present also in the structure are intramolecular aromatic C—H···N and C—H···O interactions (Table 1). A similar structure has been peviously been reported (Paulis *et al.*, 2006).

### Experimental

A mixture of 3-hydrazinylbenzonitrile hydrochloride (1.96 g, 0.01 mol) and ethyl 3-oxo-3-phenylpropanoate (1.92 g, 0.01 mol) in acetic acid (50 mL) was heated under reflux for 1.5 h, then poured into ice water. The precipitated product was filtered, giving the title compound as a powder. Single crystals were by obtained by room temperature evaporation of a solution in CH_2_Cl_2_–MeOH after 5 days.

### Refinement

H atoms were positioned geometrically (C—H = 0.95–0.98 Å) and refined using a riding model, with *U*_iso_(H) = 1.2*U*_eq_(C).

### Crystal data

**Table:**

C_16_H_11_N_3_O	*F*(000) = 544
*M**_r_* = 261.28	*D*_x_ = 1.359 Mg m^−^^3^
Monoclinic, *P*2_1_/*c*	Mo *K*α radiation, λ = 0.7107 Å
*a* = 7.6683 (3) Å	Cell parameters from 1663 reflections
*b* = 17.8013 (7) Å	θ = 3.0–29.1°
*c* = 9.7574 (4) Å	µ = 0.09 mm^−^^1^
β = 106.506 (4)°	*T* = 293 K
*V* = 1277.05 (9) Å^3^	Block, orange
*Z* = 4	0.34 × 0.30 × 0.28 mm

### Data collection

**Table:**

Agilent Xcalibur diffractometer with an Eos CCD detector	2608 independent reflections
Radiation source: Enhance (Mo) X-ray Source	1686 reflections with *I* > 2σ(*I*)
Graphite monochromator	*R*_int_ = 0.023
Detector resolution: 16.0874 pixels mm^-1^	θ_max_ = 26.4°, θ_min_ = 3.0°
ω scans	*h* = −9→9
Absorption correction: multi-scan (*CrysAlis PRO*; Agilent, 2011)	*k* = −10→22
*T*_min_ = 0.967, *T*_max_ = 1.000	*l* = −12→11
5145 measured reflections	

### Refinement

**Table:**

Refinement on *F*^2^	Secondary atom site location: difference Fourier map
Least-squares matrix: full	Hydrogen site location: inferred from neighbouring sites
*R*[*F*^2^ > 2σ(*F*^2^)] = 0.048	H-atom parameters constrained
*wR*(*F*^2^) = 0.119	*w* = 1/[σ^2^(*F*_o_^2^) + (0.0461*P*)^2^ + 0.0009*P*] where *P* = (*F*_o_^2^ + 2*F*_c_^2^)/3
*S* = 1.02	(Δ/σ)_max_ < 0.001
2608 reflections	Δρ_max_ = 0.14 e Å^−^^3^
182 parameters	Δρ_min_ = −0.13 e Å^−^^3^
0 restraints	Extinction correction: *SHELXL97* (Sheldrick, 2008), Fc^*^=kFc[1+0.001xFc^2^λ^3^/sin(2θ)]^-1/4^
Primary atom site location: structure-invariant direct methods	Extinction coefficient: 0.0083 (15)

### Special details

**Table:**

Geometry. All e.s.d.'s (except the e.s.d. in the dihedral angle between two l.s. planes) are estimated using the full covariance matrix. The cell e.s.d.'s are taken into account individually in the estimation of e.s.d.'s in distances, angles and torsion angles; correlations between e.s.d.'s in cell parameters are only used when they are defined by crystal symmetry. An approximate (isotropic) treatment of cell e.s.d.'s is used for estimating e.s.d.'s involving l.s. planes.
Refinement. Refinement of *F*^2^ against ALL reflections. The weighted *R*-factor *wR* and goodness of fit *S* are based on *F*^2^, conventional *R*-factors *R* are based on *F*, with *F* set to zero for negative *F*^2^. The threshold expression of *F*^2^ > σ(*F*^2^) is used only for calculating *R*-factors(gt) *etc*. and is not relevant to the choice of reflections for refinement. *R*-factors based on *F*^2^ are statistically about twice as large as those based on *F*, and *R*- factors based on ALL data will be even larger.

### Fractional atomic coordinates and isotropic or equivalent isotropic displacement parameters (Å^2^)

**Table:**

	*x*	*y*	*z*	*U*_iso_*/*U*_eq_	
O1	0.10361 (18)	0.26129 (9)	0.88140 (16)	0.0787 (5)	
N1	−0.4410 (2)	0.40574 (12)	1.0212 (2)	0.0843 (6)	
N2	0.14347 (17)	0.36652 (9)	0.75427 (15)	0.0460 (4)	
N3	0.25680 (17)	0.38881 (9)	0.67037 (14)	0.0453 (4)	
C1	−0.2203 (2)	0.45145 (12)	0.88230 (18)	0.0505 (5)	
C2	−0.2216 (2)	0.52432 (12)	0.8356 (2)	0.0588 (5)	
H2	−0.3015	0.5594	0.8547	0.071*	
C3	−0.1018 (2)	0.54437 (13)	0.7599 (2)	0.0658 (6)	
H3	−0.1013	0.5934	0.7272	0.079*	
C4	0.0173 (2)	0.49230 (12)	0.7321 (2)	0.0574 (5)	
H4	0.0964	0.5064	0.6800	0.069*	
C5	0.0196 (2)	0.41964 (11)	0.78127 (17)	0.0446 (5)	
C6	−0.1013 (2)	0.39869 (12)	0.85627 (17)	0.0502 (5)	
H6	−0.1024	0.3496	0.8888	0.060*	
C7	−0.3450 (2)	0.42714 (12)	0.9599 (2)	0.0597 (6)	
C8	0.1780 (2)	0.29424 (12)	0.8051 (2)	0.0521 (5)	
C9	0.3237 (2)	0.26591 (11)	0.7431 (2)	0.0535 (5)	
H9A	0.4313	0.2508	0.8176	0.064*	
H9B	0.2809	0.2239	0.6791	0.064*	
C10	0.3602 (2)	0.33246 (10)	0.66476 (16)	0.0413 (4)	
C11	0.4987 (2)	0.33671 (10)	0.58749 (17)	0.0415 (4)	
C12	0.6261 (2)	0.27999 (12)	0.60224 (19)	0.0518 (5)	
H12	0.6224	0.2387	0.6598	0.062*	
C13	0.7591 (2)	0.28432 (12)	0.5318 (2)	0.0574 (6)	
H13	0.8448	0.2462	0.5428	0.069*	
C14	0.7649 (2)	0.34450 (13)	0.4461 (2)	0.0622 (6)	
H14	0.8542	0.3472	0.3987	0.075*	
C15	0.6379 (2)	0.40112 (12)	0.4301 (2)	0.0627 (6)	
H15	0.6411	0.4420	0.3714	0.075*	
C16	0.5064 (2)	0.39714 (11)	0.50101 (19)	0.0527 (5)	
H16	0.4218	0.4357	0.4904	0.063*	

### Atomic displacement parameters (Å^2^)

**Table:**

	*U*^11^	*U*^22^	*U*^33^	*U*^12^	*U*^13^	*U*^23^
O1	0.0755 (9)	0.0780 (12)	0.1024 (11)	0.0167 (8)	0.0571 (9)	0.0415 (10)
N1	0.0917 (12)	0.0754 (15)	0.1115 (14)	0.0126 (11)	0.0702 (12)	0.0119 (13)
N2	0.0474 (8)	0.0476 (10)	0.0511 (9)	0.0017 (7)	0.0271 (7)	0.0071 (8)
N3	0.0486 (8)	0.0450 (10)	0.0495 (8)	−0.0003 (7)	0.0256 (7)	0.0014 (8)
C1	0.0449 (10)	0.0616 (14)	0.0504 (10)	−0.0010 (9)	0.0222 (8)	−0.0041 (11)
C2	0.0574 (11)	0.0548 (14)	0.0718 (13)	0.0049 (10)	0.0310 (10)	−0.0039 (12)
C3	0.0687 (13)	0.0503 (14)	0.0912 (14)	0.0033 (10)	0.0436 (12)	0.0046 (13)
C4	0.0591 (12)	0.0523 (14)	0.0727 (13)	−0.0005 (10)	0.0380 (10)	0.0022 (11)
C5	0.0407 (9)	0.0496 (12)	0.0464 (10)	0.0001 (8)	0.0171 (8)	−0.0023 (9)
C6	0.0515 (10)	0.0528 (13)	0.0532 (11)	−0.0004 (9)	0.0264 (8)	0.0018 (10)
C7	0.0591 (12)	0.0620 (15)	0.0681 (12)	0.0062 (10)	0.0345 (10)	−0.0010 (12)
C8	0.0482 (10)	0.0575 (14)	0.0555 (11)	0.0037 (9)	0.0227 (9)	0.0151 (11)
C9	0.0534 (11)	0.0523 (13)	0.0606 (12)	0.0079 (9)	0.0257 (9)	0.0132 (11)
C10	0.0420 (9)	0.0424 (11)	0.0408 (9)	0.0002 (8)	0.0141 (7)	−0.0007 (9)
C11	0.0414 (9)	0.0431 (11)	0.0420 (9)	−0.0018 (8)	0.0153 (7)	−0.0044 (9)
C12	0.0559 (11)	0.0524 (13)	0.0503 (10)	0.0055 (9)	0.0203 (9)	−0.0023 (10)
C13	0.0493 (11)	0.0630 (15)	0.0632 (12)	0.0073 (10)	0.0214 (10)	−0.0177 (12)
C14	0.0557 (12)	0.0697 (16)	0.0726 (14)	−0.0102 (10)	0.0366 (10)	−0.0184 (13)
C15	0.0710 (13)	0.0543 (14)	0.0783 (14)	−0.0056 (11)	0.0464 (11)	0.0027 (12)
C16	0.0563 (11)	0.0451 (12)	0.0658 (12)	0.0016 (9)	0.0319 (9)	0.0011 (10)

### Geometric parameters (Å, º)

**Table:**

O1—C8	1.2105 (19)	C8—C9	1.501 (2)
N1—C7	1.138 (2)	C9—H9A	0.9700
N2—N3	1.4096 (17)	C9—H9B	0.9700
N2—C5	1.417 (2)	C9—C10	1.479 (2)
N2—C8	1.377 (2)	C10—C11	1.468 (2)
N3—C10	1.289 (2)	C11—C12	1.384 (2)
C1—C2	1.374 (3)	C11—C16	1.379 (2)
C1—C6	1.382 (2)	C12—H12	0.9300
C1—C7	1.445 (2)	C12—C13	1.384 (2)
C2—H2	0.9300	C13—H13	0.9300
C2—C3	1.379 (2)	C13—C14	1.368 (3)
C3—H3	0.9300	C14—H14	0.9300
C3—C4	1.381 (2)	C14—C15	1.379 (3)
C4—H4	0.9300	C15—H15	0.9300
C4—C5	1.378 (2)	C15—C16	1.377 (2)
C5—C6	1.386 (2)	C16—H16	0.9300
C6—H6	0.9300		
			
N3—N2—C5	118.46 (15)	C8—C9—H9B	111.3
C8—N2—N3	112.64 (13)	H9A—C9—H9B	109.2
C8—N2—C5	128.83 (14)	C10—C9—C8	102.19 (15)
C10—N3—N2	107.08 (14)	C10—C9—H9A	111.3
C2—C1—C6	121.59 (16)	C10—C9—H9B	111.3
C2—C1—C7	120.86 (18)	N3—C10—C9	113.07 (14)
C6—C1—C7	117.55 (19)	N3—C10—C11	121.66 (16)
C1—C2—H2	120.7	C11—C10—C9	125.27 (15)
C1—C2—C3	118.59 (18)	C12—C11—C10	120.09 (17)
C3—C2—H2	120.7	C16—C11—C10	121.17 (16)
C2—C3—H3	119.7	C16—C11—C12	118.73 (15)
C2—C3—C4	120.6 (2)	C11—C12—H12	119.8
C4—C3—H3	119.7	C13—C12—C11	120.39 (19)
C3—C4—H4	119.8	C13—C12—H12	119.8
C5—C4—C3	120.37 (17)	C12—C13—H13	119.9
C5—C4—H4	119.8	C14—C13—C12	120.24 (18)
C4—C5—N2	120.32 (15)	C14—C13—H13	119.9
C4—C5—C6	119.48 (17)	C13—C14—H14	120.1
C6—C5—N2	120.20 (17)	C13—C14—C15	119.82 (17)
C1—C6—C5	119.32 (19)	C15—C14—H14	120.1
C1—C6—H6	120.3	C14—C15—H15	120.0
C5—C6—H6	120.3	C16—C15—C14	119.97 (19)
N1—C7—C1	177.8 (2)	C16—C15—H15	120.0
O1—C8—N2	126.74 (17)	C11—C16—H16	119.6
O1—C8—C9	128.31 (18)	C15—C16—C11	120.84 (18)
N2—C8—C9	104.95 (14)	C15—C16—H16	119.6
C8—C9—H9A	111.3		
			
C5—N2—N3—C10	177.95 (14)	C3—C4—C5—C6	1.4 (3)
C8—N2—N3—C10	0.94 (18)	N2—C5—C6—C1	179.60 (15)
N3—N2—C5—C4	−3.1 (2)	C4—C5—C6—C1	−1.0 (2)
N3—N2—C5—C6	176.29 (14)	O1—C8—C9—C10	−178.40 (19)
C8—N2—C5—C4	173.33 (17)	N2—C8—C9—C10	2.61 (18)
C8—N2—C5—C6	−7.3 (3)	C8—C9—C10—N3	−2.28 (19)
N3—N2—C8—O1	178.67 (18)	C8—C9—C10—C11	177.51 (15)
N3—N2—C8—C9	−2.32 (19)	N3—C10—C11—C12	169.70 (16)
C5—N2—C8—O1	2.0 (3)	N3—C10—C11—C16	−9.4 (2)
C5—N2—C8—C9	−178.95 (16)	C9—C10—C11—C12	−10.1 (3)
N2—N3—C10—C9	0.97 (18)	C9—C10—C11—C16	170.82 (16)
N2—N3—C10—C11	−178.83 (14)	C10—C11—C12—C13	−178.80 (16)
C6—C1—C2—C3	0.6 (3)	C16—C11—C12—C13	0.3 (3)
C7—C1—C2—C3	−178.93 (17)	C10—C11—C16—C15	179.29 (16)
C2—C1—C6—C5	0.0 (3)	C12—C11—C16—C15	0.2 (3)
C7—C1—C6—C5	179.54 (16)	C11—C12—C13—C14	−0.5 (3)
C1—C2—C3—C4	−0.2 (3)	C12—C13—C14—C15	0.1 (3)
C2—C3—C4—C5	−0.8 (3)	C13—C14—C15—C16	0.4 (3)
C3—C4—C5—N2	−179.18 (16)	C14—C15—C16—C11	−0.5 (3)

### Hydrogen-bond geometry (Å, º)

**Table:**

*D*—H···*A*	*D*—H	H···*A*	*D*···*A*	*D*—H···*A*
C4—H4···N3	0.93	2.44	2.785 (2)	102
C6—H6···O1	0.93	2.24	2.880 (3)	125
